# An autosomal dominant *ERLIN2* mutation leads to a pure HSP phenotype distinct from the autosomal recessive *ERLIN2* mutations (SPG18)

**DOI:** 10.1038/s41598-020-60374-y

**Published:** 2020-02-24

**Authors:** Jin-Mo Park, Byeonghyeon Lee, Jong-Heun Kim, Seong-Yong Park, Jinhoon Yu, Un-Kyung Kim, Jin-Sung Park

**Affiliations:** 1 Department of Neurology, Dongguk University College of Medicine, Dongguk Unversity Gyeongju Hospital, Gyeongju, Republic of Korea; 2 Department of Biology, College of Natural Sciences, Kyungpook National University, Daegu, Republic of Korea; 3 School of Life Sciences, BK21 Plus KNU Creative BioResearch Group, Kyungpook National University, Daegu, Republic of Korea; 4 Department of Neurology, School of medicine, Kyungpook National University, Kyungpook National University Chilgok hospital, Daegu, Republic of Korea

**Keywords:** Genetics, Motor neuron disease

## Abstract

Hereditary spastic paraplegia (HSP) is a heterogeneous inherited disorder that manifests with lower extremity weakness and spasticity. HSP can be inherited by autosomal dominant, autosomal recessive, and X-linked inheritance patterns. Recent studies have shown that, although rare, mutations in a single gene can lead to multiple patterns of inheritance of HSP. We enrolled the HSP family showing autosomal dominant inheritance and performed genetic study to find the cause of phenotype in this family. We recruited five members of a Korean family as study participants. Four of the five family members had pure HSP. Part of the family members underwent whole-exome sequencing (WES) to identify the causative mutation. As the result of WES and Sanger sequencing analysis, a novel missense mutation (c.452 C > T, p.Ala151Val) of *ERLIN2* gene was identified as the cause of the autosomal dominant HSP in the family. Our study suggests that the *ERLIN2* gene leads to both autosomal recessive and autosomal dominant patterns of inheritance in HSP. Moreover, autosomal dominant HSP caused by *ERLIN2* appears to cause pure HSP in contrast to autosomal recessive *ERLIN2* related complicated HSP (SPG18).

## Introduction

Hereditary spastic paraplegia (HSP) is a heterogeneous disease that manifests clinically as lower extremity weakness and spasticity. It is divided into two types, complicated and pure, and the complicated type shows additional neurological symptoms, such as ataxia, seizure, cognitive decline, extrapyramidal symptoms, and peripheral neuropathy^1^. Currently, more than 80 causal genes or loci have been identified and 75–80% of these are autosomal dominant, 25–30% are autosomal recessive, and 1–2% are X-linked^2^. A few studies have shown that mutations in Endoplasmic Reticulum Lipid Raft-Associated 2 (*ERLIN2*) gene cause autosomal recessive HSP and one recent study suggested that *ERLIN2* causes autosomal dominant HSP in two unrelated Caucasian families^2,3^. Here, we describe an Asian family with pure autosomal dominant HSP caused by a novel *ERLIN2* mutation that segregated among the family members.

## Results

### Clinical presentation

**Figure 1:**
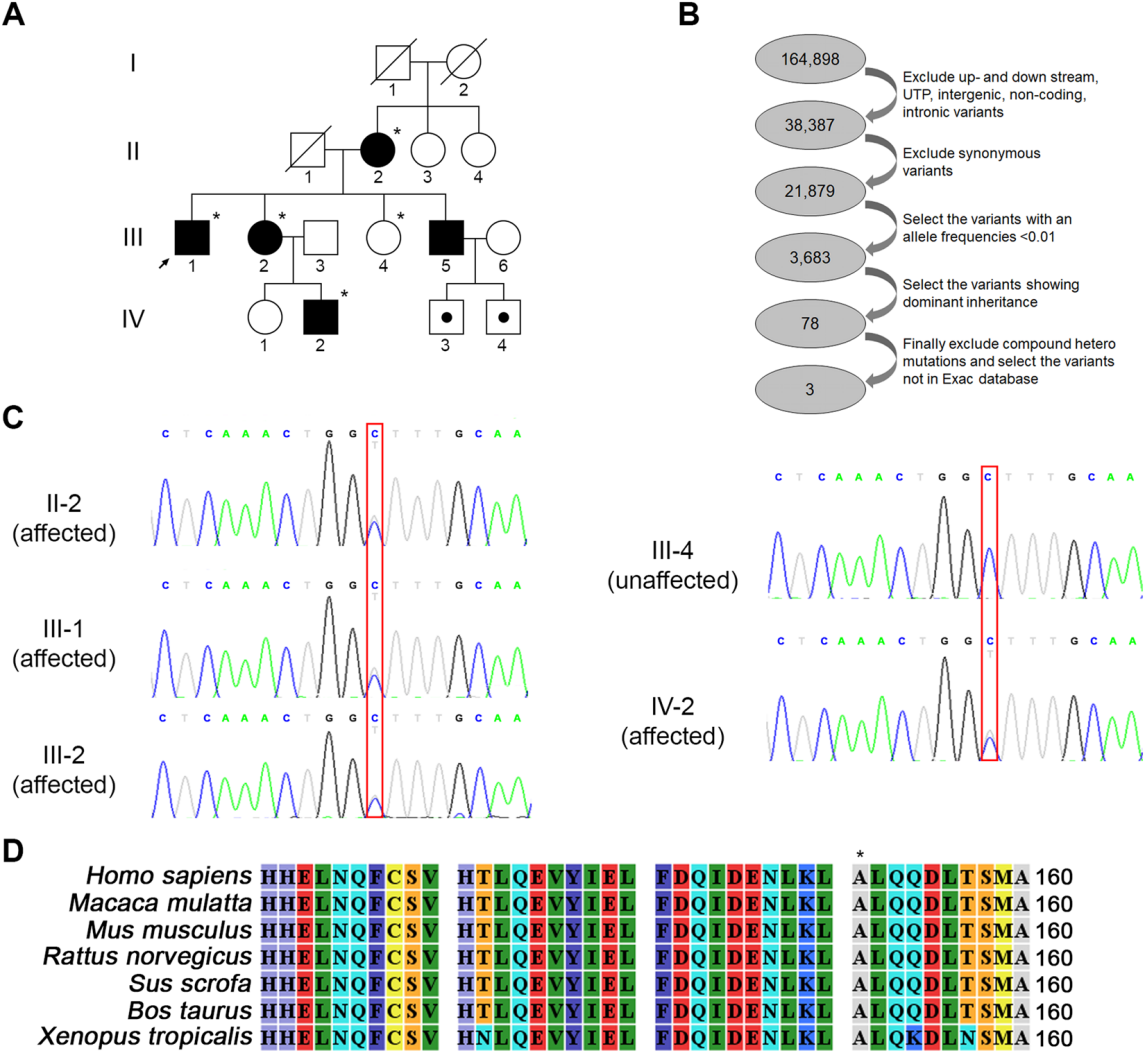
Analysis of *ERLIN2* mutation in a family. (**A**) The family pedigree is a three-generation pedigree with eight individual family members. The arrow indicates the proband and the asterisks indicate the individuals evaluated in this study. (**B**) Variants revealed by whole-exome sequencing of five family members were filtered out. The filtering strategy and the number of remaining variants at each step are shown. (**C**) The partial nucleotide sequences of exon 7 of *ERLIN2* show the c.452 C > T mutation in the affected family members (II-2, III-1, III-2, IV-2). (**D**) Multiple alignments of the ERLIN2 partial region using CLC sequence viewer 6. Amino acid sequence of human ERLIN2 was aligned with that of other species. The asterisk represents amino acid position 151 in human ERLIN2.

The proband was a 53-year-old male who presented with progressive gait disturbance. The patient noticed running difficulties as a teenager, began to use a single cane in his 30’s, and was wheelchair-bound in his 50’s. He had an autosomal dominant family history (Fig. 1A), and his mother, younger sister, and brother also experienced gait disturbance and difficulty walking. The initial neurological examination of the proband showed grade 3–4 lower limb weakness according to the Medical Research Council Scale with hyperactive deep tendon reflexes in the lower limbs, a Babinski sign, and presence of ankle clonus. The lower limb sensory system, including light and deep touch, was impaired with more significant impairment in the dorsal column. The intellectual ability of the proband was normal, he did not complain of urinary dysfunction, and his uroflowmetry results were normal. The proband had no clinical manifestations related to joint contractures, foot deformity, or scoliosis. The nerve conduction study and needle electromyography were also unremarkable. The brain magnetic resonance imaging (MRI) of the proband (III-1) did not show white matter lesions or corpus callosal thinning, and the spine MRI was normal and did not show any structural abnormality or cord atrophy.

The proband’s mother, younger sister, and younger brother (II-2, III-2, III-5) who all had HSP also showed similar neurological findings, and the details of the neurological examinations are shown (Table 1). They also showed no cognitive decline or history of seizure. The nerve conduction study and needle electromyography were performed on two additional HSP-affected individuals who were not part of the family (III-3 and III-5), and the results were unremarkable. The maternal grandparents (I-1 and I-2) who expired at 85 and 91 years of age and the mother’s siblings did not have HSP.

**Table 1:** Clinical features of patients with the *ERLIN2* mutation.

	II-2	III-1	III-2	III-5	IV-2
Sex	F	M	F	M	M
Age at examination, y	77	53	51	45	23
Age at onset, y	38	15	37	24	22
Disease duration, y	39	38	14	21	1
Symptoms at onset	Awkward gait	Gait disturbance	Awkward gait	Gait disturbance	Awkward gait
Age when wheelchair-bound	63	46	−	−	−
**Muscle weakness**					
Upper limb	5	5	5	5	5
Lower limb	2–3	3–4	4+	4+	5
**Muscle spasticity**					
Upper limb	−	−	−	−	−
Lower limb	+++	++	++	++	+
**Deep tendon reflex**					
Upper limb	1+	2+	2+	2+	2+
Lower limb	4+	4+	3+	4+	3+
Babinski sign	Bilateral	Bilateral	−/−	−/−	−/−
Ankle clonus	−/−	+/+	+/+	+/+	+/+
Bladder dysfunction	−	−	−	−	−
Sensory abnormality	+	+	+	−	−
Paresthesia	−	−	+	−	−
Foot deformity	+	−	−	−	−
Mental retardation	−	−	−	−	−
Seizure	−	−	−	−	−
Scoliosis	+	−	−	-	−

−, absent; +, mild; ++, moderate; +++, severe.

### Mutation analysis

Whole-exome sequencing was performed on the unaffected family member (III-4) and the family members with autosomal dominant HSP (II-2, III-1, III-2, IV-2; Fig. 1A). The total number of identified variants was 164,898 for the five family members. The 60.4 Mbp target region was identified by whole-exome sequencing and ≥99% of the bases had a depth of coverage of 10-fold (Table 2). To identify pathogenic variants, we filtered out variants in non-coding regions, synonymous variants, and variants with allele frequencies>0.01 using the databases 1000 genome, dbSNP, ClinVar, and Exome Aggregation Consortium (ExAC). After a family analysis of the remaining variants that showed dominant inheritance, only three variants remained (Fig. 1B). In the case of genes with variants in *CCT8* and *ZNF623*, the mutation prediction programs SIFT, FATHMM, MutationTaster, and PROVEAN showed that there wasn’t associated with the disease (Table 3). And these two genes are not associated with HSP, so we ruled out of causative gene of HSP. Of the variant genes, *ERLIN2* has been shown to cause autosomal recessive HSP^1,2,4^. We identified the c.452 C > T variant in the *ERLIN2*, but the other causative variants were not in the *ERLIN2* gene. The c.452 C > T variant was not in the 1000 genome and ExAC databases and was not found in 104 samples from normal individuals.

**Table 2:** Whole-exome sequencing statistics and coverage.

Subjects	Target regions (Mbp)	On-target yields (Mbp)	Mean depth of target regions (×)	Target coverage (%)	Coverage of target regions with depth > 10× (%)
II-2	60.4	14,019	130.9	99.7	99.1
III-1	60.4	13,498	128.5	99.9	99.2
III-2	60.4	14,393	133.7	99.7	99.1
III-4	60.4	12,674	117.4	99.7	99.0
IV-2	60.4	13,818	127.3	99.9	99.3

**Table 3:** Prediction of the pathogenic effect of candidate variants.

Gene	Reference sequence	Sequence variation	Protein variation	SIFT score	FATHMM score	MutationTaster rank score	PROVEAN score
*ERLIN2*	NM_007175.7	c.452 C > T	p.A151V	0.036 (Damaging)	−3.55 (Damaging)	0.708 (Disease-causing)	−3.24 (Damaging)
*CCT8*	NM_006585.3	c.1537 A > G	p.N513D	0.896 (Tolerated)	−1.12 (Tolerated)	0.708 (Disease-causing)	−0.95 (Neutral)
*ZNF623*	NM_014789.3	c.364 G > C	p.A122P	1.0 (Tolerated)	2.85 (Tolerated)	0.101 (Neutral)	2.99 (Neutral)

The c.452 C > T variant was confirmed to be in the exon 7 of the *ERLIN2* gene by Sanger sequencing. The HSP-affected family members were heterozygous for the c.452 C > T variant, whereas the unaffected family member only had the *ERLIN2* reference sequence suggesting on the co-segregation of genotype and phenotype within the family (Fig. 1C). The c.452 C > T single nucleotide variant resulted in an alanine to valine substitution at amino acid position 151 (p.Ala151Val) within the stomatin/prohibitin/flotillin/HflK/C(SPFH) domain of *ERLIN2*. The p.Ala151Val showed a high pathogenic effect score by the prediction programs indicating on the genetic cause of HSP in the family (Table 3),and also the p.Ala151Val is located in a region that is highly conserved in other species (Fig. 1D).

## Discussion

HSP is a heterogeneous disease that can be clinically divided into pure and complex types. Pure or uncomplicated spastic paraplegia is characterized by progressive lower limb weakness and spasticity with minimal diminution of vibratory sense. Complicated spastic paraplegia is characterized by progressive spastic weakness in the lower extremities with additional neurological symptoms, such as seizure, ataxia, cognitive decline, extrapyramidal symptoms, and peripheral neuropathy. SPG18 is a rare form of autosomal recessive complicated HSP that is characterized by early childhood onset, severe intellectual disability, joint contractures and thin corpus callosum in the brain imaging^4,5^. It is caused by *ERLIN2* mutation which has been shown to play an important role in the endoplasmic reticulum-associated degradation pathway that degrades misfolded proteins^6^. Currently, most of the literature describes autosomal recessive, complicated HSP that begins at an early age, but Rydning *et al*. showed that a novel mutation in *ERLIN2* caused autosomal dominant HSP in two North European families^3^. In accordance with Rydning *et al*., the clinical features of the family members with HSP and the *ERLIN2* mutation that we evaluated in this study are very similar to the European cases. All of the patients had only the pure symptoms of HSP and showed progressive lower limb weakness and spasticity with a mild dorsal column sensory abnormality. The age of onset of HSP for the patients in this study was in accordance with the age of onset for the European cases (range 9–46 years). It is noteworthy to state that although onset age differed among European family members, clinical severity showed no significant relationship to gender or disease duration. More studies are needed to elucidate the effect of gender but our study indicated that HSP symptoms were milder and had a later onset in female patients (II-1, III-2) than in male patients. Furthermore, longer disease duration correlated with the clinical severity, which differ from the previous cases. This reflects broad intrafamilial variability and more studies are anticipated to understand it.

Previous studies showed extremely early onset of clinical symptoms with additional neurological symptoms, such as seizure and congenital hip dislocation, cognitive decline, and joint contractures, which are frequently observed in complicated, autosomal recessive HSP^4,5,7^. In contrast, our study and the Rydning *et al*. study showed that pure, autosomal dominant HSP leads to a remarkably late onset of mild clinical symptoms.

Rydning *et al*. found an autosomal dominant, pure HSP mutation in the SPFH domain of *ERLIN2* (c.386 G > C, p.Ser129Thr), and the novel mutation we identified is also in the SPFH domain (c.452 C > T, p.Ala151Val). *ERLIN2* and *ERLIN1* are homologous genes that code for lipid raft-associated proteins that localize to the endoplasmic reticulum. They are important for ubiquitin-proteasome-mediated degradation of endoplasmic reticulum proteins and calcium signaling during lipid biosynthesis^6^.

Although our study identified that *ERLIN2* causes autosomal dominant HSP, the causal pathogenic mechanism of *ERLIN2* in autosomal recessive and autosomal dominant HSP remains unknown. We postulate that specific variants in the SPFH domain display dominant negative effects. Currently, only a few HSP types, including SPG3A, SPG7, SPG9, SPG30, SPG58, and SPG72, have been shown to have both autosomal dominant and autosomal recessive patterns of inheritance^8^. The studies on these HSP types also extrapolated possible dominant negative effects and variable penetrance patterns that require further experimental confirmation. We should also be aware of the possibility of second mutations in deep intronic regions and double trouble caused by a second mutation, but because we found domain-specific mutations in both Caucasian and Asian populations, these possibilities are reduced. We showed that a family carried a novel missense mutation in the *ERLIN2* gene that led to an autosomal dominant inheritance pattern and a pure form of HSP. In addition to a few previously identified HSP types that show both autosomal dominant and autosomal recessive inheritance, the *ERLIN2* variant should also be considered a pure form of HSP regardless of the inheritance pattern.

## Materials and Methods

### Patients and clinical assessments

This study included six members of a three-generation Korean family, and five of the six family members had HSP (Fig. 1A). We obtained approval to conduct this study from the Institutional Review Board of Kyungpook National University Chilgok Hospital (KNUCH 2018–03–006), and we obtained written informed consent from all of the study participants. All research was conducted in accordance with relevant guidelines and regulations.

Neurological examinations of motor and sensory impairments, spasticity, deep tendon reflexes, and muscle atrophy were performed. Flexor and extensor muscle strength were assessed manually using the Medical Research Council Scale. The proband underwent a nerve conduction analysis, electromyography, and brain and spine MRI to rule out structural and other neurologic disorders. The other participants underwent the routine nerve conduction analysis.

### Whole-exome sequencing

Genomic DNA was extracted from blood samples using the FlexiGene DNA kit (Qiagen, Hilden, Germany) and performed whole-exome sequencing as previously described^9^. The SureSelect V6-post kit (Agilent Technologies, Santa Clara, CA, USA) and 10 μg of genomic DNA were used for library construction. The captured DNA fragments were sequenced by Hiseq. 2500 (Illumina, San Diego, CA, USA). Data were mapped to the reference human genome, hg19, from the University of California, Santa Cruz (GRCh37 from NCBI, Feb. 2009) using BWA (Ver. Bwa-0.7.12), Picard (Ver. Picard-tools-1.130), GATK (Ver. GATKv3.4.0), and SnpEff (Ver. SnpEff_v4.1 g). The genetic variants were annotated using databases from 1000 genome release phase 3 (http://www.internationalgenome.org), dbSNP build 142 and ClinVar release 05/2015 from the National Center for Biotechnology Information (NCBI, http://www.ncbi.nlm.nih.gov), and the NHLBI ExAC (Version 0.3.1, http://exac.broadinstitute.org/). Pathogenic possibility of candidate genes were confirmed by several prediction programs, such as SIFT^10^, FATHMM (Ver. 2.3, http://fathmm.biocompute.org.uk), MutationTaster^11^, PROVEAN (v1.1.3, http://provean.jcvi.org).

### Sanger sequencing

Sequences of *ERLIN2* were confirmed by Sanger sequencing as described^12^. All exons and exon-intron boundaries of the *ERLIN2* gene were analyzed using polymerase chain reaction (PCR)-amplified direct sequencing. Specific primers were designed using Primer3 v0.4.0 (http://bioinfo.ut.ee/primer3–0.4.0/). To remove unincorporated nucleotides and primers, PCR products were digested with shrimp alkaline phosphatase (USB, Cleveland, OH, USA) and exonuclease I (USB). Purified PCR products were direct sequenced using the BigDye Terminator v3.1 cycle sequencing kit (Applied Biosystems, Foster City, CA, USA) and the 3130xl genetic analyzer (Applied Biosystems). Sequencing data and amino acid sequences were analyzed using ABI sequencing analysis software v5.2 (Applied Biosystems), Chromas pro v1.5 (Technelysium Pty Ltd., Tewantin, QLD, Australia) and CLC Sequence viewer 6 (CLC bio, Aarhus, Denmark). Sequencing data from study participants were compared to *ERLIN2* (NM_007175.7) reference sequence in the NCBI database (http://blast.ncbi.nlm.nih.gov).
